# Effects of Low Sulfur Fuel and a Catalyzed Particle Trap on the Composition and Toxicity of Diesel Emissions

**DOI:** 10.1289/ehp.7059

**Published:** 2004-07-07

**Authors:** Jacob D. McDonald, Kevin S. Harrod, JeanClare Seagrave, Steven K. Seilkop, Joe L. Mauderly

**Affiliations:** ^1^Lovelace Respiratory Research Institute, Albuquerque, New Mexico, USA; ^2^SKS Consulting Services, Siler City, North Carolina, USA

**Keywords:** diesel exhaust, emissions reduction, health effects, metals, organic carbon, particulate matter health effects

## Abstract

In this study we compared a “baseline” condition of uncontrolled diesel engine exhaust (DEE) emissions generated with current (circa 2003) certification fuel to an emissions-reduction (ER) case with low sulfur fuel and a catalyzed particle trap. Lung toxicity assessments (resistance to respiratory viral infection, lung inflammation, and oxidative stress) were performed on mice (C57Bl/6) exposed by inhalation (6 hr/day for 7 days). The engine was operated identically (same engine load) in both cases, and the inhalation exposures were conducted at the same exhaust dilution rate. For baseline DEE, this dilution resulted in a particle mass (PM) concentration of approximately 200 μg/m^3^ PM, whereas the ER reduced the PM and almost every other measured constituent [except nitrogen oxides (NO_x_)] to near background levels in the exposure atmospheres. These measurements included PM, PM size distribution, PM composition (carbon, ions, elements), NO_x_, carbon monoxide, speciated/total volatile hydrocarbons, and several classes of semi-volatile organic compounds. After exposure concluded, one group of mice was immediately sacrificed and assessed for inflammation and oxidative stress in lung homogenate. Another group of mice were intratracheally instilled with respiratory syncytial virus (RSV), and RSV lung clearance and inflammation was assessed 4 days later. Baseline DEE produced statistically significant biological effects for all measured parameters. The use of low sulfur fuel and a catalyzed trap either completely or nearly eliminated the effects.

In response to regulatory pressure aimed at decreasing the health hazards of engine emissions, diesel engine exhaust (DEE) is changing rapidly as a result of engine and fuel modifications and emissions-reduction (ER) technologies. The most drastic changes to DEE are yet to come, as fuel and ER technologies are implemented to meet the particulate matter (PM) and nitrogen oxide (NO_x_) regulatory benchmarks in 2007 and 2010 [U.S. Environmental Protection Agency (EPA) 2000]. A wide range of engine (e.g., fuel injection, combustion chamber), fuel (e.g., decreased sulfur, aromatic content), and after-treatment (e.g., particle traps, oxidation catalysts, catalyzed traps) technologies are being developed to meet these more-stringent emissions standards. Although these changes will certainly decrease regulated emissions, it is not clear how health hazards might change from historically understood DEE (U.S. EPA 2002).

With changes in DEE composition come new challenges for measurement of emissions (Durbin et al. 2003) and determination of health hazards. There is a need to understand more about the composition of exhaust produced from these emerging technologies and the resulting health benefits as the emissions change. The composition of emissions affects toxicity, as has been demonstrated by differences in the *in vitro* (bacterial mutagenicity) and *in vivo* (lung responses to instilled material) responses among seven samples of engine emissions collected from “normal-emitting” and “high-emitting” gasoline and diesel vehicles (Seagrave et al. 2002). In another study, the bacterial mutagenicity of PM collected from exhaust generated using “old” and “new” technology fuels showed decreased mutagenicity with the new fuel composition (Bagley et al. 1996). Certainly new engineering controls will change the composition of tailpipe emissions, and it is important to ensure that the new technologies provide health benefits but not produce unintended health hazards. There is a need to evaluate both the change [relative to baseline (uncontrolled) DEE emissions] in composition and health hazard of emissions as new technologies emerge.

Most of the work addressing health hazards of new ER technologies has been limited to *in vitro* assays (primarily bacterial mutagenicity) of exhaust sample extracts. Typically, samples collected to do this work only account for a small fraction of exhaust (e.g., PM), and that fraction may not accurately represent the physical, and perhaps not even the chemical, composition of the exhaust as it exists in the environment. Moreover, *in vitro* and *in vivo* assays have been shown to provide quite different rankings of toxicity among engine exhaust samples of different composition (Seagrave et al. 2003). *In vivo* responses are considered more relevant to human health hazards, and exposure by inhalation is considered the “gold standard” for hazard assessment (Driscoll et al. 2000).

In the present study, we compared a baseline case of DEE to that of a single ER case (low sulfur fuel/catalyzed ceramic trap), both generated from a “model” small-scale engine system previously shown to produce exhaust having an environmentally relevant composition (McDonald et al. 2004a). The study combined detailed characterization of the exposure atmosphere with measurements of pulmonary proinflammatory responses, heme oxygenase (HO-1) up-regulation, and resistance to infection with respiratory syncytial virus (RSV). Although we did not address the full range of health concerns related to DEE, we did include indices of the responses to acute exposure we found to be most sensitive. HO-1 is a stress-response enzyme that has been implicated as an indicator of oxidant-induced lung injury (Choi and Alam 1996; Morse and Choi 2002) and has been shown to be induced *in vitro* by DEE PM extracts (Li et al. 2002) and ambient PM extracts (Li et al. 2002, 2003). RSV is the most common cause of respiratory infection in young children (Collins et al. 2001); it can infect immune-compromised older individuals (Mlinaric-Galinovic et al. 1996); and we previously observed diminished clearance (and increased inflammation) of RSV at low exposure levels of DEE (30 μg/m^3^) generated either with a multicylinder engine (Harrod et al. 2003b) or with the single cylinder engine used in the present study (Harrod et al. 2003a).

ER markedly reduced nearly all of the measured components (both PM and gases) of the exhaust and diminished all toxicity observed with baseline DEE. These findings suggest that the use of low sulfur fuel and a catalyzed trap should markedly reduce certain health hazards and provide encouragement that ER technology will provide substantial public health benefits.

## Materials and Methods

This study included two separate inhalation exposures (termed DEE and DEE + ER, conditions summarized in Table 1) conducted at the same dilution ratio (620:1). This dilution ratio was determined by the dilution required to obtain 200 μg/m^3^ PM for the baseline DEE, which is not the minimum concentration for which we have observed effects (for RSV infection), but it is a concentration for which strongly significant effects have been reported to occur (Harrod et al. 2003a, 2003b). We conducted measurements of the biological responses and composition of the exposure atmospheres identically for the two exposures as described below.

### Exhaust generation.

The exhaust generation/exposure system has been described previously (McDonald et al. 2004a). Briefly, DEE was produced by a 5500-watt single cylinder diesel engine generator (Model YDG 5500E; (Yanmar, Osaka, Japan) that contains a 406-cc displacement air-cooled engine. Engine oil (15/40-weight, Rotella T, Shell, Houston, TX) was changed immediately prior to each 1-week exposure. The baseline DEE was generated used number 2 diesel certification fuel (Phillips Chemical Company, Borger, TX) and a high engine load condition. This fuel represented current (circa 2003) national average on-road diesel fuel. Eleven 500-watt halogen lights provided a constant load targeted at the rated capacity of the generator (5,500 watts, corresponding to 9 horsepower/3,600 rotations/min) for both exposures. For DEE + ER, the engine was operated with low sulfur fuel and an in-line catalyzed ceramic trap in the exhaust line. The low sulfur fuel/trap combination is necessary (for both large and small scale) because sulfur is detrimental to the performance and lifetime of the trap. We used a commercially available trap specifically designed for abatement of exhaust from diesel generators (fca060w4cn30; Clean Air Systems Inc., Santa Fe, NM). The trap and catalyst technologies were similar to those manufactured by the same company for larger on-road and off-road applications. Because these traps are used with low sulfur fuel, we used a prototype ultra-low sulfur diesel fuel (ECD1; provided by BP, Naperville, MD) in the ER case. The characteristics of both fuels are shown in Table 2. To ensure efficient operation, the trap was maintained at a minimum of 300°C for the duration of the exposure by thermostatically controlled heat tape. This temperature was recommended by the manufacturer, and it has been used for other DEE trap studies with multicylinder engines (e.g., SAE 1998).

### Exposure system and exposure atmosphere.

The animal exposure chamber was a 1-m^3^ whole-body inhalation chamber (Hazleton H-1000; Lab Products, Maywood, NJ) operated at a flow rate (250 L/min) that produced approximately 15 air exchanges/hr. Temperature, relative humidity, and flow (orifice plate mated to electronic pressure transducer) were monitored and recorded at all times. Temperature was maintained between 22 and 26°C. Exposures were conducted 6 hr/day (~ 0730–1330 hours) for 7 consecutive days. DEE concentration for baseline conditions was controlled by manually adjusting the air dilution to the predetermined PM concentration. These adjustments were based on PM concentration measurements made both in “real time” and integrated over 30-min periods as described below. For DEE + ER, the catalyzed trap decreased the PM concentration sufficiently that it was not practical to use PM for system control. The trap also decreased the gases to levels below those useful for control of the system; one exception was NO_x_, which could not be used because the NO_x_ analyzer failed during the exposure study. To control the system for DEE + ER, the trap was by-passed before the exposure started and the exhaust dilution was adjusted to match the DEE test based on the concentration of carbon monoxide measured directly in the engine exhaust and exposure chamber. Once the dilution valves were set, the engine was turned off, the exposure chamber was flushed for 10 min, and the animals were placed in the chamber. The exhaust was then routed through the trap for the entire exposure period. After the exposure, the animals were removed and the dilution was measured again by the same approach to ensure that it did not change during the exposure. The DEE and DEE + ER exposures were accompanied by separate concurrent control groups exposed to filtered air (HEPA filter and charcoal scrubber). The filtered air controls were treated exactly the same as the DEE-exposed animals (including animal movement immediately before and after DEE + ER exposures).

### Exposure atmosphere characterization.

The composition of the exposure atmospheres were characterized in detail (> 250 analytes) using methods reported previously (McDonald et al. 2004a). The measurements, measurement techniques, and laboratories that conducted the analyses are summarized in Table 3. As mentioned above, during the exposure study, the NO_x_ analyzer malfunctioned; therefore, NO_x_ values are reported from measurements collected at a later date with identical fuel/engine/trap operational conditions and dilutions used during the exposures.

PM concentration, the metric we used to target the DEE dilution rate, was measured gravimetrically by sampling from the exposure chamber for 30-min intervals on 47-mm Pallflex filters (Pall-Gelman, East Hills, NY) in aluminum in-line filter holders (In-Tox Products, Inc., Albuquerque, NM). We measured pre- and postsample filter weights using a Mettler MT5 microbalance (Mettler, Columbus, OH). A static discharger was used before weighing filters to avoid any interference from electrical charge on the filters.

### Animals and husbandry.

Young (8–10 weeks of age) C57Bl/6 mice (Charles River Laboratories, Inc., Wilmington, MA) were housed under pathogen-free conditions according to Association for Assessment and Accreditation of Laboratory Animal Care (AAALAC)-approved guidelines and protocols. Routine serologic screens for mouse pathogens showed no preexisting infections in the study groups. Mice were housed in the whole-body exposure chambers and were provided water *ad libitum* at all times. Food (Harlan TEKLAD Rodent Diet; Harlan Teklad, Madison, WI) was provided *ad libitum* during nonexposure hours. At the end of the 7-day exposure period, a portion of each treatment group was infected with RSV, and the remainder was immediately sacrificed for analysis of lung inflammation and HO-1.

### Resistance to infection.

We assessed viral clearance and lung histopathology as described by Harrod et al. (2003b). Briefly, control and DEE-exposed C57Bl/6 mice (eight per group) were instilled intratracheally with 10^6^ plaque-forming units of cultured RSV immediately after the last day of exposure. Mice were housed individually in pathogen-free conditions in a designated animal room for 4 days. At 4 days postinfection, we analyzed one lobe of the lung for the presence of RSV virus by densitometric analysis of virus-specific mRNA transcripts that were isolated by gel electrophoresis after amplification(reverse transcriptase-polymerase chain reaction). RSV mRNA transcripts were ratioed to amplified β-actin (internal control) mRNA levels to account for intersample variability in mRNA isolation and amplification. RSV was thus compared in each treatment group as the average of the RSV/β-actin responses for each animal.

Lung cross-sections for histopathology of the RSV infected mice were obtained approximately 500 μm caudal to the junction of the mainstream bronchus, stained with hematoxylin and eosin, and analyzed by a pathologist under light microscopy. The pathologist scored (0–4 scale) the levels of inflammation in the airways and vessels without knowledge of the origin of the sample.

### Inflammation.

We measured inflammatory signaling proteins (cytokines) in homogenates of the right caudal and middle lung lobes (six per group). Immediately after sacrifice, lungs were frozen in 1 mL Dulbecco’s phosphate-buffered saline (PBS) with a cocktail of proteinase inhibitors. Before analysis, lungs were removed from the freezer and brought to room temperature. Lungs were homogenized for 1 min at full speed in a Tissuemizer (Tekmar, Mason, OH) and centrifuged for 5 min at 14,000 × *g*. The supernatant was transferred to clean microfuge tubes and kept on ice. Cytokines [tumor necrosis factor-α (TNF-α), interferon -γ (IFN-γ), interleukin-6 (IL-6)] were determined (two measurements for each cytokine for each sample) by ELISA using commercially available mouse analysis kits (Biosource International, Camarillo, CA). To normalize the cytokine measurements to total protein, supernatants were diluted to 2 mg/mL in PBS and total protein was assayed by the Coomassie-dye binding assay (Pierce, Rockford, IL) with bovine serum albumin as the standard.

### HO-1.

We measured HO-1 induction in lung homogenate by Western blotting using 30 μg of a sample of lung homogenate supernatant (prepared as described above for inflammatory indicators) in 1× Laemmli sample buffer containing 25 mM dithiothreitol. Samples were heated for 5 min at 95°C and resolved on a 15% polyacrylamide gel. Proteins were electroblotted to polyvinylidene difluoride membranes. The blots were blocked with 5% nonfat dry milk in Tris-buffered saline with 0.1% Tween-20, and incubated with 1 μg/mL polyclonal anti-HO-1 (Calbiochem, San Diego, CA) followed by 1 μg/mL horse-radish peroxidase-labeled goat anti-rabbit IgG. HO-1 was detected by chemiluminescent (ECL, Amersham, Piscataway, NJ) exposure of BioMax Film (Kodak, Rochester, NY) and quantified by densitometry as described by Li et al. (2003).

### Statistical analysis.

We used analysis of variance (ANOVA) to evaluate DEE and DEE + ER responses relative to values from concurrent control groups. Levene’s test (Levene 1960) was first performed to evaluate the appropriateness of the standard ANOVA assumption of equality of variances among experimental group responses. These tests showed that for all end points except lung histopathology, there was significant evidence of inequality of variances (*p* < 0.05). To address this problem, we used a weighted least-squares analysis (Neter et al. 1996) using the reciprocals of the variances in experimental groups as weights. F-test contrasts (Searle 1971) were used to compare DEE and DEE + ER responses with baseline values in concurrent control groups. Because the baseline values for the two control groups differed substantially for some end points, reported means and SEs were scaled by the mean values from concurrent control groups. Statistical significance was assessed at *p* = 0.05 and *p* = 0.01; however, several treatment groups showed *p*-values much lower than this. Calculations were performed using SAS software (SAS Institute, Cary, NC).

## Results

### Exposure characterization.

Table 4 summarizes the composition, reporting the concentrations of major components (by mass) and several composite (summed) subclasses of material along with select organic compounds, primarily those designated as hazardous air pollutants by the U.S. Environmental Protection Agency. (Data for individual compounds are available from the corresponding author upon request.) The percent change in the concentration of each component or component class after ER implementation is also shown in Table 4. Data are reported here as average concentrations of multiple (2–3) samples that were collected during the exposure period.

**Figure 1:**
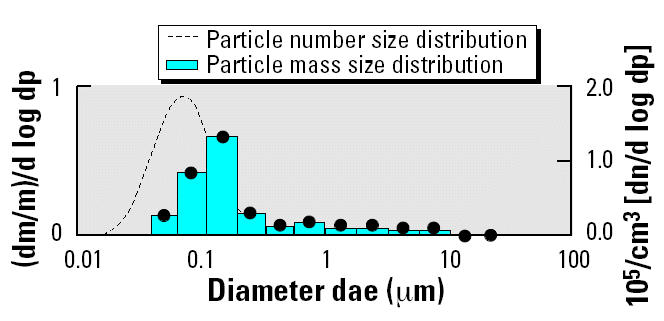
Particle mass and number size distribution in the DEE exposure atmosphere. Abbreviations: ae, aerodynamic diameter; d, derivative; m, mass; p, particle diameter; n, number. Control and DEE + ER particle mass and number concentrations are not shown because they were too low to measure accurately.

ER significantly reduced the concentration of nearly every component. Most constituents (except NO_x_, select elements, and the carbonyls) were in the range of the background concentrations observed in the control exposure chamber. NO_x_ (~ 98% nitrogen monoxide and 2% nitrogen dioxide, both with and without ER) reductions were not expected, as the manufacturer of the catalyzed trap (Clean Air Systems Inc.) reported modest to no reduction in NO_x_ concentrations in their product description. ER reduced the PM and particle number concentration so that it was too low to accurately measure particle size or particle number (< 10^3^ particles/cm^3^). The particle number and mass distributions for the baseline DEE are illustrated in Figure 1, which shows a distribution having a mass median aerodynamic diameter of 110 nm and a particle number median diameter of 80 nm. The majority of the other components in the ER atmosphere were also reduced. Most of these reductions were > 60% relative to baseline DEE; there was no detectable black (elemental) carbon and reduced particle organic carbon content. Similar to the control atmosphere, the PM component of the ER atmosphere was nearly 100% organic carbon. Small amounts of the elements, especially calcium and zinc, which are lube oil and fuel additives (Docekal et al. 1992), were observed in DEE and decreased substantially with ER.

The ER atmosphere had low quantities of both gases and PM, many of which were in the same range as the concentrations observed in the control atmosphere. However, several individual organic compounds were present in DEE and DEE + ER at concentrations significantly above background. Acetylene, a compound that has been used in ambient source apportionment studies as an indicator of mobile source emissions, was enriched in DEE but reduced in DEE + ER to background levels, as was also true for most of the aromatic [including polycyclic aromatic hydrocarbons (PAHs)] and alkene (including 1,3-butadiene) compounds. However, removal of the carbonyl compounds, especially formaldehyde and acetaldehyde, was much less efficient (~17–45%).

### Lung toxicity.

**Figure 2:**
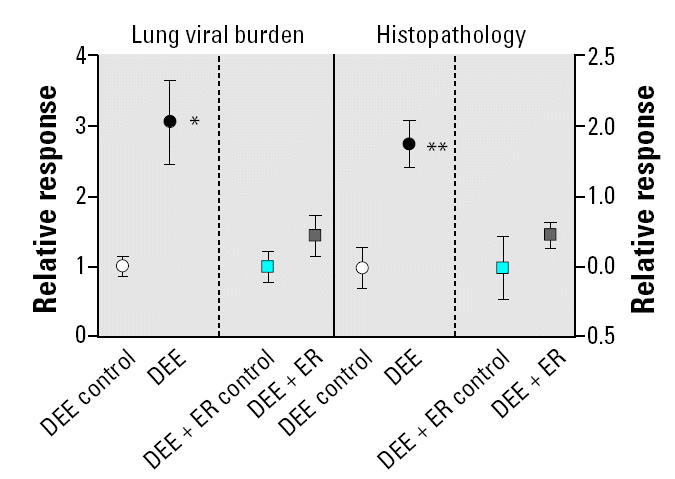
Viral retention and lung histopathology in RSV-infected mice exposed to either clean air (DEE or DEE + ER control), DEE, or DEE + ER. Error bars indicate SE. DEE and DEE + ER exposures were conducted at equivalent dilutions.
*p = 0.002 and **p = 0.003 compared to control.

**Figure 3:**
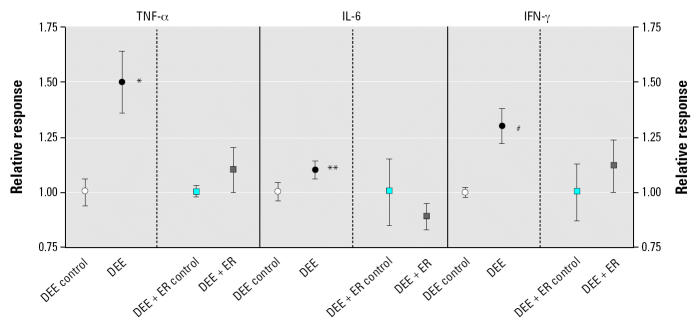
Inflammatory signaling proteins measured in lung homogenates of mice exposed to clean air (DEE control, DEE + ER control), DEE, or DEE + ER. Error bars indicate SE. DEE and DEE + ER exposures were conducted at equivalent dilutions.
*p = 0.003, **p = 0.036, and ^#^*p* = 0.001, compared to control.

**Figure 4:**
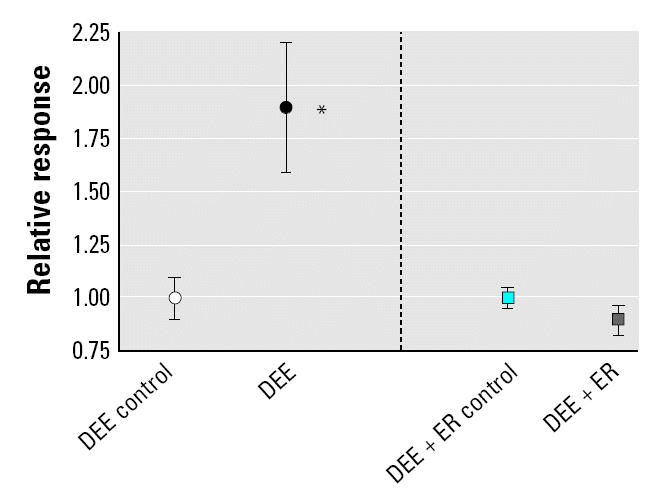
HO-1 measured in lung homogenates of mice exposed to clean air (DEE Control, DEE + ER Control), DEE, or DEE + ER. Error bars indicate SE. DEE and DEE + ER exposures were conducted at equivalent dilutions.
*p = 0.003.

The results of the cytokine/HO-1 up-regulation in noninfected animals and the lung viral burden/histopathology scores after exposure/RSV infection are shown in Figures 2–4. The DEE exposure resulted in statistically significant differences from control exposed animals for all measured lung responses, but the DEE + ER exposure resulted in no significant differences from control for any biological measurement. Figure 2 summarizes the lung viral burden and lung histopathology of virus-infected mice. As expected from previous studies (Harrod et al. 2003a, 2003b), DEE exposure significantly (*p* = 0.002) decreased the clearance of virus from the lung and significantly increased(*p* = 0.003) the histopathology scores. Figure 3 shows the increase in cytokines. All cytokines were significantly elevated above control values in the DEE group, but not in the ER group. HO-1, the oxidative stress response indicator, also significantly increased after DEE exposure but not after DEE + ER exposure (Figure 4).

## Discussion

The present study showed that implementation of a low sulfur fuel/catalyzed trap combination decreased the concentration of most components of emissions and diminished the biological effects of DEE on viral clearance, inflammation, and oxidative stress. These findings suggest that this type of ER technology may have substantial health benefits. Of course, ER technologies may vary considerably, and it is not known how broadly these results might apply to other technologies.

The ER case significantly decreased nearly every measured exposure constituent except NO_x_ to background levels. Except for a few volatile organic compounds and elements, the ER and control exposure atmosphere had similar low concentrations of both gases and PM. These similarities suggest that a portion of the constituents observed in the DEE exposure atmosphere downstream of the trap was contributed by background in the dilution air or by the rodents themselves. Although the dilution air was pretreated by filtration through HEPA and charcoal filters, these filters do not efficiently remove CO or methane. The contribution of rodent respiration and excretion to the composition whole-body exposure atmospheres has been discussed previously (McDonald et al. 2004b). Among the compounds that are contributed by respiration and background are the C_2_-C_12_ alkanes, for which there were similar concentrations among all of the exposure atmospheres (including DEE). Similar to the control chamber, the small PM component of the DEE + ER exposure atmosphere was nearly 100% organic carbon, which was likely contributed by the rodents (dander, exhaled organics, etc.).

Despite the contribution of rodents and dilution air to the exposure atmospheres, several individual organic compounds were present in DEE and DEE + ER at concentrations significantly above background, indicating a variable efficiency of removal. Acetylene, a compound that has been used in ambient source apportionment studies as an indicator of mobile source emissions, was enriched in DEE but reduced in the DEE + ER to background levels. This also occurred for most of the aromatic (including PAH) and alkene (including 1,3-butadiene) compounds. However, removal of the carbonyl compounds, especially formaldehyde and acetaldehyde, was much less efficient (~ 17–45%). These findings agree with previous reports comparing a baseline DEE to DEE with low sulfur fuel and a trap (Durbin et al. 2003), where the ER was most efficient at removing acetylene, moderately efficient at removing alkenes/aromatics, and poor at removing volatile carbonyls.

Although it provided an important first look at the effects of ER, this study had several limitations. First, the exhaust was not produced by an engine that would be used on-road. We previously demonstrated the usefulness of this model system by showing both similar composition (McDonald et al. 2004a) and similar biological responses (Harrod et al. 2003a) at selected operating conditions compared to DEE produced from a multicylinder diesel engine operated on a heavy-duty engine cycle. This model system was therefore considered adequate to show “proof of concept” or to develop testing protocols. However, the applicability of the present results to emissions generated from larger on-road and off-road engine systems needs to be confirmed. In addition, it may be important to assess the performance of a wider range of ER technologies operating under a variety of engine operation conditions. The high constant workload and new particle trap (emissions may change after trap “ages”) used in this study allowed the optimal performance of the ER. Under this condition, the emissions were substantially decreased. Rudell et al. (1996) reported that humans exposed to DEE from an idling vehicle both with and without a ceramic particle trap (no catalyst) had inflammation (as assessed by increase in neutrophils and infiltration of alveolar macrophages into their airways) in both cases. In that study the ceramic trap removed only half of the particle count.

Although the results of the present study clearly demonstrated a near total mitigation of the effects of DEE exposure on retardation of viral clearance and pathology, inflammation, and oxidative stress, the results must be extrapolated to humans with caution. There is no direct evidence for the effect of DEE on human resistance to RSV infection, although RSV is certainly a pervasive human pathogen (Collins et al. 2001); proximity to heavy traffic has been associated with increased categories of respiratory illnesses that encompass viral infection (e.g., Romieu et al. 2002). The correspondence between responses of mice and humans can be questioned, but the use of mice as models for the pathophysiology of human RSV infection is widely accepted (Graham et al. 1988). The use of only one exposure concentration for DEE was another limitation of this study; however, we previously demonstrated that the effect of DEE inhalation on RSV clearance was concentration related (Harrod et al. 2003b). We believe that the single concentration, which is in the range of occupational exposures to DEE (e.g., McDonald et al. 2002) was adequate to explore the effects of the ER strategy.

The induction of respiratory inflammation by exposure to whole, diluted DEE and its partial mitigation by a particle trap (also at single concentrations) has been demonstrated in humans (Rudell et al. 1996). Although evidence for the role of oxidative stress in responses to DEE is derived largely from animal and *in vitro* studies, the induction of HO-1 stress response protein has been well documented in humans as an indirect indicator of oxidative stress (Morse and Choi 2002). In this study we did not attempt to fully characterize the nature and magnitude of DEE-induced oxidative damage.

The approach used in this study had the advantage of being *a*) by inhalation, *b*) short-term, and *c*) relevant to known public health hazards. It provided data using contemporary chemical and physical characterization techniques coupled to three biological response categories that are relevant to human health end points observed by laboratory (e.g., Rudell et al. 1996;  Sydborn 2001) and epidemiology studies (e.g., Nicolai et al. 2003; Romieu et al. 2002; Samet et al. 2000; Van Vliet et al. 1997). The present study illustrates one approach to the challenge posed to the scientific and regulatory community to develop appropriate testing protocols aimed at placing changing DEE health hazards in context. We did not assess several classes of health effects that may be of importance (e.g., tumor formation, cardiovascular toxicity, exacerbation of asthma/inflammation), including effects that are commonly studied after long-term exposure periods. The study included only a few of the biological responses that have been reported in response to DEE, but these are among the most sensitive (e.g., RSV end points respond to DEE diluted as low as 30 μg/m^3^). The concordance in response among the biological end points lends confidence in the overall conclusions of decreased health hazard.

## Conclusions

ER (low sulfur fuel/catalyzed trap) technology decreased or diminished the emissions and the toxicity of DEE. With ER in place there was no detectable black (elemental) carbon, particle organic carbon in the range of background air, and decreased (relative to uncontrolled emissions) concentrations of the elements. Nearly all-gaseous components (except NO_x_, which was only slightly reduced, and select carbonyls) were in the range of background air. Baseline DEE exposures (no emission controls) produced significant biological responses in all measured end points. These responses, including lung inflammation (response to lung injury), resistance to a viral infection, and induction of a lung oxidative stress indicator, were not observed with ER in place. These results indicate that the use of low sulfur fuel and a catalyzed trap markedly reduce the DEE health hazard associated with resistance to infection, inflammation, and oxidative stress.

## Figures and Tables

**Table 1:** Summary of exposure atmosphere generation test conditions.

	Engine operation	Fuel	After-treatment	Dilution target
DEE	High load	No. 2 Cert	None	200 μg/m^3^ PM
DEE + ER	High load	ECD1	Catalyzed trap	Same dilution as DEE

No. 2 Cert, number 2 diesel certification fuel.

**Table 2:** Properties of the number 2 diesel certification fuel (No. 2 Cert) and the ECD1 low sulfur fuel.

	No. 2 Cert	ECD1
API gravity	35.8	35.3
Specific gravity 60/60	0.85	0.85
Viscosity	2.4	2.8
Sulfur (ppm)	371	14
Aromatics (volume %)	29	32
Cetane index	47.6	46.1
Cetane number	47.3	47.7

API gravity is an arbitrary scale representing the gravity of liquid petroleum; cetane number is a measure of ignition quality of diesel fuel; and cetane index is an approximation of cetane number based on the API gravity and mid-boiling point of a fuel.

**Table 3:** Summary of measurements, measurement conditions, and analytical techniques used to characterize exposure atmosphere composition.

Analysis	Collection device	Collection media	Sample flow rate (L/min)	Analytical instrument
Particle mass	Aluminum in-line filter holder	TIGF filter	10	MB
NO_x_	Chemiluminescence analyzer	NA	0.4	NA
CO	Photoacoustic analyzer	NA	1	NA
Organic/elemental carbon*^a^*	Aluminum in-line filter holder	Quartz filter	20	TOR
Ions (sulfate/nitrate/ammonium)*^a^*	Aluminum in-line filter holder	Quartz filter	20	IC
Metals and other elements*^b^*	Teflon in-line filter holder	Teflon filter	20	ICPMS
Speciated organic compounds				
Volatile hydrocarbons (C_1_–C_12_) *^a^*	Volatile organic sampler	Electropolished canister	0.1	GCFID
Volatile carbonyls	Volatile organic sampler	DNPH cartridge	0.3	LC/UV
Semivolatile/aromatic/alkane	Filter/PUF sampler	TIGF filter/PUF/XAD/PUF XAD-4/PUF	60	GCMS
Size distribution				
0.05–10 μm particle mass distribution	MOUDI impactor*^c^*	Aluminum	30	MB
0.02–0.7 μm particle number distribution	SMPS	NA	0.3	NA

Abbreviations: DNPH, dinitrophenylhydrazine; GCFID, gas chromatography flame ionization detection; GCMS, gas chromatography/mass spectrometry; IC, ion chromatography; ICPMS, inductively coupled plasma mass spectrometry; LC/UV, liquid chromatography/ultraviolet detection; MB, microbalance; NA, not applicable; PUF, polyurethane foam; SMPS, scanning mobility particle sizer; TIGF, Teflon impregnated glass fiber; TOR, thermal/optical reflectance; XAD, XAD resin.

a Analyses conducted at the Desert Research Institute, Reno, NV.

b Analyses conducted at the Carlsbad Environmental Monitoring and Research Center, Carlsbad, NM.

c Source: MSP Corp, St. Paul, MN.

**Table 4:** Comparative composition of DEE, DEE + ER, and control (clean air) exposure chambers.

Analyte or chemical class	Control	DEE	DEE + ER	DEE vs. DEE + ER (percent decrease)
NO_x_*^a^* (ppm)	< 0.04	2.1	1.9	10
Nonmethane volatile organic (μg/m^3^)	54.4	162.3	63.2	61
CO (ppm)	0.3	2.0	0.2	90
Particle mass (μg/m^3^)	5.1	235.7	7.0	99
Particle composition				
Black (elemental) carbon (μg/m^3^)	0.0	200.3	0.0	100
Organic carbon (μg/m^3^)	4.5	39.9	4.2	90
Nitrate (μg/m^3^)	0.5	0.2	0.0	100
Sulfate (μg/m^3^)	0.2	0.0	−0.1	NA
Ammonium (μg/m^3^)	0.0	−0.1	−0.1	NA
Sum of elements (μg/m^3^)	0.0	2.1	0.7	67
Speciated organic classes				
Sum carbonyl (μg/m^3^)	5.3	37.7	21.9	42
Acetylene (alkyne) (μg/m^3^)	0.5	16.7	0.4	98
Sum of C_2_–C_12_ alkanes (μg/m^3^)	26.6	27.7	21.1	24
Sum of C_2_–C_12_ alkenes (μg/m^3^)	3.0	31.7	1.6	95
Sum of volatile aromatics (μg/m^3^)	8.5	25.2	13.2	48
Sum of C_15_–C_30_ alkanes (μg/m^3^)	6.8	26.7	9.6	64
Sum of naphthalenes (μg/m^3^)	1.0	4.7	1.0	80
Sum of phenanthrenes (μg/m^3^)	0.5	6.2	0.4	93
Sum of other SVOC PAHs (μg/m^3^)	0.4	1.7	0.6	65
Sum of particle PAHs (ng/m^3^)	0.0	23.0	0.0	100
Sum of Oxy-PAHs (μg/m^3^)	0.05	1.29	0.08	94
Select speciated organics				
Formaldehye (μg/m^3^)	1.8	14.1	11.6	17
Acetaldehyde (μg/m^3^)	1.5	17.0	9.4	45
Benzaldehyde (μg/m^3^)	0.5	1.9	0.3	84
Ethene (μg/m^3^)	0.5	25.9	0.5	98
1,3-Butadiene (μg/m^3^)	0.0	2.2	0.0	100
Benzene (μg/m^3^)	0.4	4.5	0.2	95
Pyrene (μg/m^3^)	0.03	0.34	0.02	93
Benzo[*a*]pyrene (ng/m^3^)	0.00	0.08	0.00	100
Dibenzothiopene (μg/m^3^)	0.06	0.10	0.05	43
9-Fluorenone (μg/m^3^)	0.05	1.07	0.05	95
Xanthone (μg/m^3^)	0.00	0.12	0.00	100
Select elements				
Zinc (μg/m^3^)	−0.01	0.71	0.07	90
Calcium (μg/m^3^)	−0.03	0.41	0.22	47
Iron (μg/m^3^)	−0.02	0.24	0.07	71
Potassium (μg/m^3^)	−0.01	0.16	0.04	73
Silicon (μg/m^3^)	−0.09	0.26	0.07	73
Magnesium (μg/m^3^)	0.00	0.08	0.03	58
Copper (μg/m^3^)	0.01	0.06	0.05	11
Lead (μg/m^3^)	0.01	0.07	0.02	74

Abbreviations: PAHs, polycyclic aromatic hydrocarbons; SVOC, semivolatile organic compound.

a Concentrations not obtained during exposures due to analyzer failure; data was obtained from an identical fuel and engine operation exposure study.
